# Quality of Life of Hemodialysis Patients in Greece: Associations with Socio-Economic, Anthropometric and Nutritional Factors

**DOI:** 10.3390/ijerph192215389

**Published:** 2022-11-21

**Authors:** Ioanna Floria, Ioanna Kontele, Maria G. Grammatikopoulou, Theodoros N. Sergentanis, Tonia Vassilakou

**Affiliations:** 1 Department of Public Health Policy, School of Public Health, University of West Attica, 196 Alexandras Avenue, 11521 Athens, Greece; 2 Department of Rheumatology and Clinical Immunology, Faculty of Medicine, School of Health Sciences, University of Thessaly, Biopolis, 41110 Larissa, Greece

**Keywords:** hemodialysis, Mediterranean Diet, quality of life, eating habits, obesity, education

## Abstract

Chronic kidney disease (CKD) is a serious public health problem that, in recent decades, has taken on significant dimensions with serious effects on the quality of life (QoL) of patients. The purpose of this cross-sectional study is to evaluate the QoL of a sample of hemodialysis patients in Greece and the possible correlations with socio-economic and anthropometric factors, as well as with adherence to the Mediterranean Diet (MD). During September–November 2019, one-hundred and five (*n* = 105) patients with end-stage CKD (63.4 ± 13.09 years of age) who were regularly monitored in five public and private hemodialysis units in the region of Attica, completed a demographic questionnaire, the MedDietScore questionnaire, and the KDQOL-SF questionnaire. Females presented worse QoL than males (*p <* 0.05), and older patients presented worse QoL than younger patients (*p <* 0.01). Patients of higher educational status presented better QoL scores than those of lower educational status (*p <* 0.01), while those with low financial status presented lower QoL scores than patients of middle and high financial status (*p <* 0.01). Obese patients had lower QoL scores than overweight patients (*p <* 0.05), and overweight males scored higher than normal weight males (*p <* 0.05). Age was negatively correlated to the total and most of the scales of QoL (*p <* 0.01). A majority of the patients (90.5%) showed a moderate adherence to MD, although “work status” was the only QoL scale that was correlated to MD. Age, educational status and financial status accounted for 28.1% of the variance in the KDQOL-SF total score. Hemodialysis patients need support in various levels, such as social, financial and educational, as well as nutritional counseling to adopt a balanced diet and maintain a healthy weight, in order to achieve a better quality of life.

## 1. Introduction

Chronic kidney disease (CKD) is a serious medical condition with a significant public health burden, as it is associated with increased morbidity and mortality [1]. More than 800 million individuals worldwide suffer from CKD, with prevalence being higher in women than men, in older individuals, and in patients with diabetes and hypertension [2]. Renal Replacement Therapy (RRT) is necessary for patients with end-stage CKD, and, according to the European Renal Association (ERA) Registry’s 2019 Annual Report, the treatment modality at the start of RRT is hemodialysis (HD) for 84% of the patients, peritoneal dialysis (PD) for 11%, and kidney transplantation for 5% [3]. Although RRT increases survival rates, the 5-year unadjusted patient survival probability for patients commencing HD or PD is only 42.3%, indicating the exceptional sensitivity of these patients compared to the general population [3].

The World Health Organization defines quality of life as “an individual’s perception of their position in life in the context of the culture and value systems in which they live and in relation to their goals, expectations, standards and concerns” [4]. The term quality of life (QoL) refers to all the physical, mental, social and economic parameters of a person’s life and is influenced by a variety of factors, such as demographic, medical, and psychological [5]. Health and QoL are very closely related to each other, and for this reason, when examining the QoL of people suffering from chronic diseases it is essential to examine the Health-Related Quality of Life (HRQOL). HRQOL is a multidimensional concept defined as the individual’s subjective assessment of the impact of the disease and its treatment on the physical, psychological and social level, assessing the impact on their daily functioning and well-being [6]. The concept of HRQOL is the result of the interaction between the patient’s life conditions and the way in which these conditions are perceived by the patient himself [7].

Patients on hemodialysis develop symptoms that affect their daily activities with serious effects on functionality, but also on their QoL. Recent studies indicate that the QoL of patients on hemodialysis is significantly lower than that of control groups [8,9]. Hemodialysis affects patients’ QoL physically, psychologically, and socially, as their daily life changes dramatically due to the frequent hemodialysis sessions [8]. Patients undergoing a dialysis session may experience complications or symptoms during the session, such as hypotension, cramps, nausea, vomiting, headache, pruritus, precardiac pain, pericarditis and fever with chills [10]. Complications, symptoms and clinical problems are also experienced after the sessions and cause functional limitations in the physical state of health [10]. Patients on hemodialysis have a greater possibility to present depressive symptoms when compared to the general population and to other patients [11]. Hemodialysis sessions are frequent, affecting the patient’s socialization, and are associated with loss of autonomy and increased dependence on others to help them perform their daily activities. Many patients cannot continue working and quit their jobs, which may make them even more socially distant [8]. The QoL of the patients is also associated with many social factors, such as having a spouse, having private or public health care, educational level, age, and finally the existence or lack of a family environment [8].

For hemodialysis patients, lifestyle changes are very important to improve their health and well-being. One important component of these changes is their daily eating habits [12]. Studies show that a large percentage of patients with end-stage CKD show evidence of malnutrition [13]. Malnutrition is a strong predictor of disease outcome for up to five years, but it is also associated with frailty, increased risk of infections, and low QoL [14].

The Mediterranean Diet (MD) has been recognized as one of the healthiest dietary patterns, consisting of a variety of healthy foods and ensuring the high consumption of fruits, vegetables, nuts, fish, legumes, cereals and olive oil, and low intakes of meat and processed foods [15]. High MD adherence has been associated with a longer life expectancy and a protective role against the main non-communicable diseases, such as cardiovascular diseases, type 2 diabetes, metabolic syndrome and cancer [16,17,18,19,20]. Moreover, it has been shown that high MD adherence is associated with a lower incidence of chronic kidney disease and with better survival rates in CKD patients [21,22]. A higher adherence to MD has also been associated with better QoL in the general population [23,24], but also in patients with diabetes [25] and breast cancer survivors [26]. To our knowledge, no studies have examined the association of MD adherence and QoL in hemodialysis patients.

The purpose of the current cross-sectional study is to evaluate the QoL of patients with end-stage chronic kidney disease who undergo renal function replacement therapy with hemodialysis, and its correlation with socio-economic factors and with the adherence to the MD pattern. The main objectives were:

(a) To explore the main socio-economic factors that are linked to the level of QoL of hemodialysis patients. It was hypothesized that a lower financial and a lower educational status will be linked to lower QoL, while patients that are unmarried or divorced would have lower QoL than married ones.
(b) To explore the level of adherence of the patients to the MD pattern, and to explore the association of the adherence to the total QoL and to the different scales. It was hypothesized that a higher MD adherence would be associated with better QoL.
(c) To examine if other personal characteristics, such as age and BMI status, are associated with QoL. We hypothesized that a higher age and higher BMI will be associated with lower QoL.

## 2. Materials and Methods

### 2.1. Participant Recruitment

Data collection took place between September and November 2019. Adult CKD patients on hemodialysis participated in the study. Patients were regularly monitored in four public (Hippokrateio Hospital, Evangelismos Hospital, Gennimatas Hospital and Hellenic Navy Hospital) and one private (White Cross Clinic) hemodialysis units in the region of Athens, Attica, Greece. The sample was drawn at random from patients attending scheduled hemodialysis sessions at the above units.

Initially, the directors of the hemodialysis units were informed regarding the survey’s purpose and methodology. Then, the heads of the units informed the patients about the study’s content, purpose, their voluntary participation, and finally, the protection of their personal data. Finally, the questionnaires were distributed to all patients by the main researcher before the initiation of their hemodialysis session. The patients were asked to complete the questionnaires by themselves and return them to the researcher before the end of their session. The main researcher assisted every person who was unable to complete them alone.

Exclusion criteria for participation in the study involved not speaking the Greek language and the poor health status of some patients, who were unable to complete the questionnaires.

### 2.2. Ethical Permission, Consent and Anonymity

Approval for the current study was granted by each hospital’s scientific bioethics committee (Hippokrateio Athens Hospital n.180/24 September 2019, Evangelismos General Hospital n.576/14 October 2019, Hellenic Navy Hospital n.7/19/27 September 2019, Gennimatas General Hospital n.31387/25 October 2019). The patients received oral and written information regarding their anonymity, their voluntary participation, the protection of their personal data, as well as the possibility of withdrawing at any time. Only the patients that signed the relevant consent form were allowed to participate in the study. Patients’ personal data were coded in order to ensure the protection of the provided information.

### 2.3. Questionnaires and Tools Applied

#### 2.3.1. Personal Characteristics

The first section of the questionnaire included information regarding the gender, age, family status, financial status and educational status of the participants. Moreover, current body height, current body weight before hemodialysis, and current dry body weight after hemodialysis were self-reported. Patients were asked to report the body weight that was measured the same day they completed the questionnaire. Patients’ weight is routinely recorded by the units’ nurses every time before and after the hemodialysis session, using electronic (digital) scales calibrated to 0.1 kg. The body mass index (BMI) was then calculated for each participant as dry body weight (kg), divided by height (m2). Patients were classified as underweight (BMI < 18.5 kg/m2), normal weight (BMI 18.5–24.9 kg/m2), overweight (BMI 25.0–29.9 kg/m2) and obese (BMI ≥ 30 kg/m2).

#### 2.3.2. Adherence to the Mediterranean Diet

In order to evaluate the adherence to the Mediterranean Diet, the MedDietScore was used. MedDietScore is an index that estimates the adherence level to the traditional MD pattern. It includes questions regarding the frequency of consumption of nine food groups (non-refined cereals, potatoes, fruits, vegetables, legumes, fish, red meat and products, poultry, full fat dairy products), as well as the use of olive oil in cooking and consumption of alcoholic beverages. Scores of 0, 1, 2, 3, 4 and 5 were assigned for each answer. Specifically, for the consumption of food groups that are considered preferable according to the MD pattern (non-refined cereals, potatoes, fruits, vegetables, legumes, fish, olive oil) the score was greater as the consumption was more frequent. For the consumption of foods that are considered not compatible with the MD pattern (red meat and products, poultry, full fat dairy products) a reverse scale was assigned. Regarding alcohol consumption, a score of 5 was assigned for the consumption of less than 300 mL/day, scores of 1–4 for consumption of 600–700 mL/day, 500–600 mL/day, 400–500 mL/day and 300–400 mL/day, respectively, and a score of zero was assigned for the consumption of more than 700 mL/day, as well as for none. The total score ranged from 0 to 55, with 0–11 indicating very low adherence, 12–22 low adherence, 23–33 moderate adherence, 34–44 high adherence and 45–55 very high adherence to the MD [27].

#### 2.3.3. Quality of Life of Hemodialysis Patients

The patients’ QoL was evaluated with the Kidney Disease Quality of Life Short Form Questionnaire (KDQOL-SF). The inventory was used to evaluate the health-related QoL of patients on hemodialysis, and the initial version included 134 questions. A short version with 80 questions was later developed, with 43 of the questions relating to the kidney disease, and 36 concerning general information regarding the patient’s health. The short version was divided in four sections: patient’s health, patient’s kidney disease, effects of kidney disease on his/her daily life and satisfaction with care. The items produced 19 scales: physical functioning (10 items), role physical (4 items), pain (2 items), general health (5 items), emotional well-being (5 items), role emotional (3 items), social function (2 items), energy/fatigue (4 items), symptoms (12 items), effects of kidney disease (8 items), burden of kidney disease (4 items), work status (2 items), cognitive function (3 items), quality of social interaction (3 items), sexual function (2 items), sleep (4 items), social support (2 items), dialysis staff encouragement (2 items) and patient satisfaction (1 item) [28,29]. The short form of the inventory was translated and validated in Greek studies [30].

### 2.4. Statistical Analyses

Analyses were performed using SPSS version 22.0 (SPSS Inc., Chicago, IL, USA). For all analyses, the level of statistical significance was set at 0.05. Continuous variables are presented as mean ± standard deviation and categorical variables are presented as frequencies and percentages.

For all categorical variables, such as the participants’ socio-economic characteristics, a Chi-squared test was performed. Independent samples’ t-tests were used to examine the differences regarding the anthropometric characteristics between males and females, and the scores of the questionnaires between the two sexes. Moreover, comparisons were made between patients under and over the age of 63.4 years old, which is the mean age of the sample of the study. Due to the relatively small number of participants, it was decided to only compare two age groups, and not divide the sample into more age groups. One-way ANOVA with Bonferroni post-hoc analyses was performed to examine the differences in KDQOL-SF scores between the different socio-economic and BMI statuses. Pearson’s correlation coefficient (r) was used to investigate linear associations among the examined variables. Finally, multiple stepwise backward linear regression analysis was performed to investigate the independent predictors of the total QoL score. To facilitate the regression analysis, socio-economic variables were dichotomized.

## 3. Results

### 3.1. Population Characteristics

Initially 120 questionnaires were distributed to patients. Eight patients refused to take part in the survey and seven patients did not return the questionnaires. A total of 105 patients (*n* = 105) were included in the analyses. Of these, 68 (64.8%) were men and 37 (35.2%) were women. The mean age of the participants was 63.4 ± 13.1 years, with no significant differences between men and women. As it was expected, men were significantly taller and heavier than women, although no significant differences regarding BMI were observed (Table 1).

The socio-economic characteristics of the participants are presented in Table 2. No significant differences were found between males and females regarding their family and financial status. However, there was a significant difference regarding their educational status (Χ^2^_(2)_ = 10.73, *p* = 0.005), with more women than men having only completed primary education.

### 3.2. Adherence to Mediterranean Diet

The mean score on the MedDietScore questionnaire was 27.52 ± 3.88, with no significant difference between men and women, and the majority of participants fell in the category of moderate adherence to MD (Table 3). Moreover, when the MedDietScore was compared between different age group categories—namely patients under 63.4 years and over 63.4 years—it was found that younger patients had a higher score (28.27 ± 4.01) than older patients (26.81 ± 3.65), although this difference is not statistically significant (*p* = 0.054). Finally, no significant differences on MedDietScore were found regarding the different family, educational and financial statuses, nor on the different BMI categories.

### 3.3. Quality of Life

Regarding the total score on the KDQOL-SF questionnaire, as it is shown in Table 4, a significant difference between males and females was observed, with females having lower scores (p = 0.037). Females had also significantly lower scores in the scales of physical function (p = 0.041), symptoms (p = 0.003) and effects of kidney disease (p = 0.017). When the different age groups were compared, it was found that older patients have a significantly lower overall score of QoL (p = 0.007), as well as significantly lower scores in the scales of physical function (p = 0.000), role physical (p = 0.003), role emotional (p = 0.007), social function (p = 0.003), sexual function (p = 0.014) and sleep (p = 0.010).

The total scores on the KDQOL-SF questionnaire were also examined according to the socio-economic status, as well as the BMI status of the patients (Table 5). Family status wasn’t associated with the total QoL score in the overall sample and in the separate groups. Educational status was found to be a significant factor, as participants that have tertiary education have a significantly higher total QoL score, when compared to patients with primary (*p* = 0.006) and secondary (*p* = 0.008) education. This association was also significant in the group of males (*p* = 0.032) and in the group of patients under 63.4 years old (*p* = 0.020), but not in females and patients over 63.4 years old. Regarding financial status, it was found that patients of low financial status have a significantly lower total QoL score than patients of middle (*p* = 0.004) and high (*p* = 0.003) financial status. The same finding was statistically significant in females (*p* = 0.045), in the younger patients’ group (*p* = 0.042) and in the group of older patients (*p* = 0.023), but not in males. Finally, BMI status was associated with total QoL score. Due to the small number of underweight patients, they were combined with the normal weight patients for the analysis. It was found that obese patients have significantly lower scores of QoL than overweight patients (*p* = 0.014), but not significantly lower than those of normoweight patients. In the females’ group, no significant differences were found between the BMI groups. However, in the males’ group, overweight patients scored higher than obese patients (*p* = 0.022), but also higher than normal weight patients (*p* = 0.017). In the group of patients under 63.4 years old, obese patients had significantly lower scores than overweight patients (*p* = 0.015). No difference was found in the group of patients over 63.4 years old.

### 3.4. Correlations and Regression Analysis

In the overall sample, negative correlations were found between age and the total score of KDQOL-SF (r = −0.394, *p* ≤ 0.01), the general health scale (r = −0.223, *p* ≤ 0.05), the physical function scale (r = −0.500, *p* ≤ 0.01), the role physical scale (r = −0.323, *p* ≤ 0.01), the role emotional scale (r = −0.358, *p* ≤ 0.01), the social functioning scale (r = −0.312, *p* ≤ 0.01), the bodily pain scale (r = −0.261, *p* ≤ 0.05), the cognitive function scale (r = −0.271, *p* ≤ 0.01), the quality of social interaction scale (r = −0.233, *p* ≤ 0.05), the symptoms scale (r = −0.224, *p* ≤ 0.05), the sexual function scale (r = −0.464, *p* ≤ 0.01) and the sleep scale (r = −0.350, *p* ≤ 0.01). Moreover, the MedDietScore was significant correlated only with the work status scale (r = 0.212, *p* ≤ 0.05), but there was no significant correlation with the total QoL score, or with any other QoL scale (Table 6).

Finally, a multiple linear regression model with a backward elimination method was used to identify the predictors of the KDQOL-SF total score. As detailed in Table 7, age, educational status and financial status accounted for 28.1% of the variance in the KDQOL-SF total score (adjusted R^2^ = 0.281, F_(3,100)_ = 14.438, *p* < 0.001).

## 4. Discussion

The present study examined the quality of life of patients on hemodialysis in Greece, and explored its association with socio-economic, anthropometric and nutritional factors.

According to the results of this investigation, female patients on hemodialysis have lower QoL scores than male patients, regarding the total score, as well as the scales of physical function, symptoms, and effects of kidney disease. Previous studies have also indicated that women are more vulnerable than men, regarding the effects of chronic kidney disease and hemodialysis on their QoL. A study in Taiwan found that women on hemodialysis have lower self-reported health-related QoL, especially regarding depression-related scales [31], while in a study in Romania female patients on hemodialysis scored significantly lower than males in the scales of physical functioning and vitality [32]. More recent studies in Brazil, Iran and India have also found that women score lower in the majority of the scales of QoL [33,34,35]. It has been hypothesized that this difference between males and females may be explained by women’s multiple domestic tasks and responsibilities that cannot be avoided by prioritizing the demands of their illness [32]. On the other hand, there are studies that have found better or equal QoL of females when compared to males [36,37].

Another significant finding of the current study is that QoL is associated with the patients’ age. Older patients had significantly lower scores in total QoL and in most of the scales, when compared to younger patients, with differences being significant in the scales of physical function, role physical, role emotional, social function, sexual function and sleep. Moreover, regression analysis showed that the QoL total score decreases as the age of the patients increases. Negative correlations between age and QoL in hemodialysis patients have been found in a number of previous studies [5,35,37,38,39,40]. Bohlke et al. (2008) have suggested that this association can be explained by the fact that older patients have more comorbid conditions [40]. On the other hand, van Loon et al. (2017) found that, although patients ≥ 75 years old have significantly lower scores on physical functioning scales compared to the <65 years group, older age was associated with higher scores in emotional health [41]. Respectively, Sethi et al. (2021) revealed that patients over 60 years old have better QoL scores in the social domain [35], while Greene et al. (2005) observed that increasing age was associated with higher scores in most of the QoL scales, and suggested that older patients have a greater level of comfort regarding their health and social life [42].

Regarding the socio-economic factors that affect the QoL of hemodialysis patients, family, educational and financial status were examined. Educational status was positively correlated with the QoL scores, with patients with tertiary education having better QoL than those with primary and secondary education. This positive association has also been found in a significant number of studies [5,8,36,37,43]. One possible explanation is that patients with a higher level of education have wider access and understanding to information about the disease, the treatment, and the prevention of complications than patients with a lower level of education. Moreover, patients with better education often have jobs that require less physical effort [8]. However, it is interesting that Bayoumi et al. (2013) have found a positive association of education with most of QoL scales, but a negative association with the satisfaction with medical care. Researchers attributed this finding to the fact that patients with higher education are more aware of their rights and of the quality of medical services, and consequently may be more demanding and critical [37].

Financial status was another indicator that was positively correlated with QoL, as was expected. This result is in accordance to the recent findings of Sethi et al. (2021), who found that the QoL scores were directly positively related to the monthly family income [38]; likewise, with the findings of Jesus et al. (2019) who reported that higher income level was associated with higher scores in the environmental domain [8]. Pakpour et al. (2010) also found that poor mental health is associated with lower economic status [39]. Additionally, El-Habashi et al. (2020) indicated that income was associated with all scales of QoL, assuming that higher income would lead to better self-esteem, feeling of satisfaction, and fewer worries about the future [44]. Lower QoL in patients of lower financial status may also be associated with the high cost of treatments and the difficulty of continuing to work. Moreover, a better financial status gives access to better medical services and better health behaviors (such as healthy nutrition and physical activity) [8].

Family status wasn’t associated with the total QoL score in the current study, in contrast to the results of recent studies that have shown that being married is linked to better QoL scores—especially in the social function scales—compared to being unmarried or divorced [8,35]. Nevertheless, no association between family status and QoL has also been indicated in other studies [37,44], with the researchers attributing this result to the strong family ties in their culture. Indeed, it can be hypothesized that this also applies to the current study, as in Greece the members of paternal families continue to keep strong bonds, and a patient, even unmarried, usually has help from his parents or siblings.

Regarding the anthropometric factors, BMI status was found to be associated with the total QoL score, although BMI was not a significant predicting factor in the regression model. In the total sample of the current study obese patients presented significantly lower scores of QoL than overweight patients, and male overweight patients scored significantly higher than obese and normal weight patients. Although BMI and QoL association has not been thoroughly examined in hemodialysis patients, there are studies that agree with the finding of inverse correlation between these two variables [36,45,46]. Bossola et al. (2009) found that obese patients score lower than normal-weight patients on the physical functioning scale and the physical component summary score, but did not score significantly lower on the scale related to mental health [47]. The finding of higher QoL scores in the group of overweight patients, compared to normal weight patients, could be explained by the “obesity paradox” that has been consistently reported in end-stage CKD patients, where a higher BMI is paradoxically associated with better survival [46,48]. It should be noted that in the studies mentioned, as well as in the current study, the common WHO BMI standards for adults were used. However, there is evidence that WHO cut-offs may not be appropriate for older adults and some organizations suggest using age-related BMI criteria. For example, the Committee on Diet and Health in the USA proposes BMI of 24–29 kg/m^2^ as normal for people aged ≥ 65 years [49]. This should be taken into consideration in future studies of older hemodialysis patients.

Finally, one of the aims of the current study was to examine the possible associations between QoL and adherence to the MD pattern. According to our results, the majority of participants had moderate adherence to MD, as it has been found in previous Greek studies of end-stage CKD patients [50]. The MedDietScore was significantly correlated with the work status scale, which could be explained by the fact that persons with better working status have also better financial status, and therefore are more likely to have access to healthy foods that are included in the MD pattern, such as olive oil, fruits, vegetables and non-refined cereals. No correlation was found between MD adherence and total QoL or other scales of QoL. To our knowledge, this was the first study that examined the possible correlations of MD adherence to QoL on hemodialysis patients, so more studies are needed in this field.

The current study has certain limitations that should be acknowledged. First, the cross-sectional design cannot address the causality of the results. Secondly, patients that participated were only from hemodialysis units within the prefecture of Attica, and no patients from non-urban regions were included. Moreover, the number of patients was relatively small, and therefore no generalization of the results is possible. It should also be noted that the years that the patients were receiving hemodialysis were not recorded; thus, it was not possible to examine if the QoL worsens as the time of hemodialysis increases. Another significant limitation of the study is that anthropometric characteristics of the patients (height and weight) were self-reported, while there was no way to acquire additional data (such as body fat, muscle mass, waist circumference etc.). It should also be mentioned that the tools used could not examine the level of knowledge of the patients regarding the advantages of following the MD pattern, or regarding the proper diet of a patient in hemodialysis.

## 5. Conclusions

The present cross-sectional study recorded data on the quality of life of end-stage CKD patients undergoing hemodialysis. As was expected, socio-economic characteristics were found to be significantly associated with the QoL. Moreover, personal characteristics such as age and BMI status were inversely correlated to QoL, while the adherence to MD was not correlated to the total QoL.

Further research should be undertaken to explore how specific eating behaviors and adherence to eating patterns, such as MD, may influence the QoL of these patients. Moreover, interventional studies could explore the possible advantages of adopting healthy eating habits and keeping a healthy body weight.

Hemodialysis patients are a vulnerable population that needs specific medical and psychological care. The type of care should be individualized according to the specific needs of each patient. Those needs are affected by educational, financial, and family status, as well as sex, age, and anthropometric characteristics. The whole scientific team that is responsible for their care should be thoroughly informed regarding the factors that affect the patient’s QoL. Educational material regarding healthy habits that may improve QoL, such as healthy nutrition, physical activity and social support, could be useful for medical staff, patients, and their families. Patients with end-stage CKD need multidisciplinary and individualized care, so that all their needs are met and their optimal quality of life is ensured in all its domains.

## Figures and Tables

**Table 1:** Participants’ age and anthropometric characteristics (mean ± SD) (T-test).

	Total (*n* = 105)	Males (*n* = 68)	Females (*n* = 37)	t/X^2^	*p*
Age (years)	63.4 ± 13.1	62.57 ± 13.0	65.00 ± 13.27	0.907	0.367
Height (cm)	1.68 ± 0.10	1.72 ± 0.09	1.61 ± 0.06	7.528	<0.001
Body weight (kg) before hemodialysis	73.4 ± 15.7	76.8 ± 15.8	67.2 ± 13.5	3.130	0.002
Dry body weight (kg)	71.2 ± 15.4	74.5 ± 15.7	65.0 ± 12.9	0.624	0.002
BMI (kg/m^2^)	25.13 ± 4.67	25.14 ± 4.63	25.09 ± 4.81	0.045	0.964
Underweight	4 (3.8%)	2 (2.9%)	2 (5.4%)	1.147	0.766
Normal weight	54 (51.4%)	37 (54.4%)	17 (45.9%)		
Overweight	32 (30.5%)	19 (27.9%)	13 (35.1%)		
Obese	15 (14.3%)	10 (14.7%)	5 (13.5%)		

BMI: Body Mass Index.

**Table 2:** Socio-economic characteristics of the participants (frequencies and percentages).

		Total (*n* = 105)	Males (*n* = 68)	Females (*n* = 37)	X^2^	*p*
Family Status	Single	23 (21.9%)	13 (19.1%)	10 (27.0%)	2.096	0.351
	Married	61 (58.1%)	43 (63.2%)	18 (48.6%)		
	Divorced/Widower	21 (20.0%)	12 (17.6%)	9 (24.3%)		
Educational Status	Primary	22 (21.0%)	8 (11.8%)	14 (38.9%)	10.730	0.005
	Secondary	40 (38.1%)	28 (41.2%)	12 (33.3%)		
	Tertiary	42 (40.0%)	32 (47.1%)	10 (27.8%)		
Financial Status	Low	27 (25.7%)	15 (22.1%)	12 (32.4%)	1.753	0.416
	Middle	49 (46.7%)	32 (47.1%)	17 (45.9%)		
	High	29 (27.6%)	21 (30.9%)	8 (21.6%)		

**Table 3:** Scores (mean ± SD) of the participants in the MedDietScore questionnaire and MD adherence status (frequencies and percentages).

		Total (*n* = 105)	Males (*n* = 68)	Females (*n* = 37)	t/X^2^	*p*
Total MedDietScore		27.52 ± 3.88	27.62 ± 4.22	27.35 ± 3.21	0.334	0.739
MD adherence status	Low adherence	7 (6.7%)	6 (8.8%)	1 (2.7%)	1.458	0.482
	Moderate adherence	95 (90.5%)	60 (88.2%)	35 (94.6%)		
	High adherence	3 (2.9%)	2 (2.9%)	1 (2.7%)		

MedDietScore, Mediterranean Diet Score; MD, Mediterranean Diet.

**Table 4:** Scores (mean ± SD) of the participants in the KDQOL-SF and its scales according to sex and age (*t*-test).

		Sex		Age Group	
KDQOL-SF Components	Total (*n* = 105)	Males (*n* = 68)	Females (*n* = 37)	<63.4 Years Old (*n* = 51)	≥63.4 Years Old (*n* = 54)
KDQOL-SF total score	60.44 ± 17.07	62.98 ± 16.27	55.75 ± 17.71 *	65.04 ± 17.77	56.09 ± 15.30 **
General Health	42.10 ± 22.49	44.26 ± 22.23	38.14 ± 22.73	44.80 ± 24.39	39.55 ± 20.43
Physical Function	50.20 ± 31.91	54.87 ± 32.74	41.62 ± 28.82 *	63.33 ± 29.62	37.80 ± 29.12 **
Role Physical	39.04 ± 42.87	43.38 ± 42.89	31.08 ± 42.24	51.47 ± 44.83	27.31 ± 37.70 **
Role Emotional	48.25 ± 44.57	51.96 ± 44.37	41.44 ± 44.72	60.13 ± 43.21	37.03 ± 43.27 **
Social Function	63.57 ± 29.92	65.44 ± 31.30	60.13 ± 27.29	72.30 ± 30.24	55.32 ± 27.42 **
Bodily Pain	72.23 ± 32.14	76.72 ± 29.63	63.98 ± 35.24	78.33 ± 28.92	66.48 ± 34.18
Energy/Fatigue	55.66 ± 25.13	57.13 ± 24.87	52.97 ± 25.72	57.64 ± 25.32	53.79 ± 25.04
Emotional Well-Being	63.76 ± 22.40	64.92 ± 21.37	61.62 ± 24.33	64.78 ± 22.25	62.79 ± 22.70
Burden of Kidney Disease	48.58 ± 26.57	51.03 ± 27.33	44.08 ± 24.86	51.13 ± 27.83	46.18 ± 25.36
Cognitive Function	83.74 ± 20.08	84.11 ± 20.08	83.06 ± 20.34	86.40 ± 21.12	81.23 ± 18.90
Quality of Social Interaction	76.76 ± 20.98	77.64 ± 20.30	75.13 ± 22.36	79.08 ± 20.99	74.56 ± 20.92
Symptoms	80.79 ± 14.08	83.73 ± 10.55	75.37 ± 17.87 **	82.65 ± 12.78	79.03 ± 15.12
Effects of Kidney Disease	57.01 ± 19.35	60.30 ± 18.60	50.96 ± 19.49 *	58.01 ± 20.06	56.07 ± 18.79
Sexual Function	41.80 ± 35.89	43.85 ± 36.12	37.89 ± 35.42	50.52 ± 34.97	32.50 ± 34.68 *
Sleep	56.95 ± 25.03	58.38 ± 24.86	53.58 ± 25.38	63.13 ± 25.88	50.60 ± 22.80 **
Social Support	78.25 ± 27.74	81.37 ± 25.02	72.52 ± 31.72	78.75 ± 29.45	77.77 ± 26.30
Work Status	34.76 ± 32.61	37.50 ± 36.00	29.72 ± 24.88	30.39 ± 38.83	38.88 ± 25.07
Patient Satisfaction	75.80 ± 18.41	75.62 ± 19.09	76.12 ± 17.36	75.81 ± 19.23	75.78 ± 17.77
Dialysis Staff Encouragement	88.94 ± 17.09	89.36 ± 17.16	88.17 ± 17.16	87.99 ± 16.00	89.85 ± 18.18

* <0.05 ** <0.01.

**Table 5:** Scores (mean ± SD) of the participants in the KDQOL-SF, according to family, educational, financial and BMI status (one-way ANOVA).

			Sex		Age Group	
		Total (*n* = 105)	Males (*n* = 68)	Females (*n* = 37)	<63.4 Years Old (*n* = 51)	≥63.4 Years Old (*n* = 54)
Family Status	Single	60.73 ± 16.51	65.90 ± 13.43	54.00 ± 18.35	66.51 ± 12.68	44.35 ± 15.79
	Married	61.81 ± 18.03	63.10 ± 17.72	58.72 ± 18.90	67.81 ± 19.55	57.36 ± 15.65
	Divorced/Widower	56.11 ± 14.61	59.40 ± 13.85	51.73 ± 15.24	52.90 ± 17.91	58.09 ± 12.56
Educational Status	Primary	54.39 ± 20.22	56.65 ± 21.41	53.10 ± 20.21	61.08 ± 25.03	50.57 ± 16.73
	Secondary	56.82 ± 15.71	58.19 ± 15.56	53.61 ± 16.27	58.02 ± 18.15	55.62 ± 13.19
	Tertiary	67.75 ± 13.65 **	68.76 ± 13.87 *	64.50 ± 13.07	72.51 ± 11.18 *	61.98 ± 14.39
Financial Status	Low	50.38 ± 16.82 **	54.33 ± 18.63	45.45 ± 13.38 *	54.30 ± 17.30 *	47.25 ± 16.32 *
	Middle	63.19 ± 14.45	64.61 ± 11.55	60.52 ± 18.88	66.58 ± 15.96	60.43 ± 12.73
	High	65.14 ± 18.10	66.70 ± 19.03	61.04 ± 15.78	70.62 ± 18.00	57.37 ± 15.83
BMI Status	Normal weight/Underweight	59.25 ± 17.17	60.18 ± 16.68	57.34 ± 18.47	64.30 ± 18.65	53.85 ± 13.80
	Overweight	66.68 ± 15.74	72.47 ± 12.36 *	58.21 ± 16.75	72.86 ± 12.90	61.23 ± 16.35
	Obese	51.68 ± 15.38 *	55.89 ± 14.52	43.25 ± 14.86	49.18 ± 13.64 *	53.34 ± 17.03

* <0.05 ** <0.01; BMI, body mass index.

**Table 6:** Correlation between age, BMI, MedDietScore and the items of KDQOL-SF.

	**Age**	**BMI**	**MedDietScore**
KDQOL-SF total score	−0.394 **	0.046	0.069
General Health	−0.223 *	0.135	0.022
Physical Functioning	−0.500 **	0.008	0.079
Role Physical	−0.323 **	0.129	0.041
Role Emotional	−0.358 **	−0.011	0.104
Social Functioning	−0.312 **	0.093	0.022
Bodily Pain	−0.261 *	−0.079	0.092
Energy/Fatigue	−0.185	0.116	0.009
Emotional Well-Being	−0.167	0.057	0.121
Burden of Kidney Disease	−0.079	−0.036	0.044
Cognitive Function	−0.271 **	0.064	0.078
Quality of Social Interaction	−0.233 *	−0.003	−0.025
Symptoms	−0.224 *	0.002	0.014
Effects of Kidney Disease	−0.101	0.007	−0.007
Sexual Function	−0.464 **	0.108	0.066
Sleep	−0.350 **	−0.130	−0.073
Social Support	−0.013	0.110	−0.015
Work Status	0.038	0.053	0.212 *
Patient Satisfaction	−0.136	−0.019	−0.017
Dialysis Staff Encouragement	−0.061	−0.001	0.098

* <0.05; ** <0.01; BMI, body mass index; MedDietScore, Mediterranean Diet Score.

**Table 7:** Multiple regression analysis predicting the KDQOL-SF total score.

						95% CI	
Variables	B	SE	Βeta	*t*	*p*	Lower	Upper
Age (years)	−0.459	0.110	−0.349	−4.161	0.000	−0.678	−0.240
Educational Status (Tertiary vs. Primary/Secondary)	8.338	2.971	0.243	2.806	0.006	2.443	14.223
Financial Status (Middle/High vs. Low)	10.459	3.351	0.269	3.121	0.002	3.810	17.108
	Variables excluded by model: BMI, family status, sex						

CI, confidence intervals; SE, standard error.

## Data Availability

All data are available upon request to the first author.
